# The interplay between mental resilience, loneliness, and psychological distress: a cross-sectional study in “Siberia of Greece”

**DOI:** 10.3389/fpubh.2026.1772164

**Published:** 2026-02-06

**Authors:** Georgia-Nektaria Porfyri, Ioanna Kastari, Elpida Stratou, Aikaterini Gamvroula, Aikaterini Toska, Evangelos C. Fradelos, Pavlos Sarafis, Theocharis Konstantinidis, Maria Saridi

**Affiliations:** 1 Department of Psychiatry, General Hospital of Argolida, Argos, Greece; 2 Health Administration, Hellenic Open University, Patras, Greece; 3 Department of Occupational Therapy, University of West Attica, Athens, Greece; 4 Department of Nursing, University of Thessaly, Volos, Greece; 5 Department of Nursing, Hellenic Mediterranean University, Larissa, Greece

**Keywords:** anxiety, community center, depression, loneliness, meaning in life, mental resilience, stress

## Abstract

**Introduction:**

Community center recipients often face depression and anxiety, compounded by socioeconomic factors like poverty and unemployment.

**Materials and methods:**

The study surveyed 120 community center recipients in a remote area of Greece. Participants were administered a questionnaire, including socio-demographic data, the Depression Anxiety Stress Scale-21 (DASS-21), the UCLA Loneliness Scale (UCLA-LS-20), the Connor-Davidson Resilience Scale (CD-RISK-25) and the Meaning in Life Questionnaire (MLQ). Data analysis was conducted using SPSS 26.0.

**Results:**

The total scale of depression, anxiety, and stress was negatively correlated with age (*p* = 0.015), while chronic physical illness (*p* = 0.019) and feelings of loneliness (*p* = 0.003) were related to depression, anxiety, and stress. Living arrangements (*p* = 0.019) were directly correlated with meaning in life (*p* = 0.003) and mental resilience (*p* = 0.007).

**Discussion:**

Mental resilience and meaning in life were protective factors, against depression, anxiety, and stress. Loneliness was a risk factor for psychological distress, while chronic physical illnesses and living alone were risk factors for depression and loneliness. Although older age was a protective factor against psychological distress, being married was associated with anxiety. The study emphasizes that mental health is deeply intertwined with social connectivity and physical health. Interventions should prioritize reducing loneliness and fostering resilience to mitigate the impact of socioeconomic stressors. Future strategies should aim to enhance tailored psychosocial approaches by leveraging family support systems and helping individuals find meaning in their lives.

## 1 Introduction

The global burden of disease attributed to mental health conditions has reached critical proportions, with recent WHO reports estimating that over 1.1 billion people worldwide live with psychiatric morbidity (1). A significant treatment gap persists, particularly in low- and middle-income countries, where approximately 71% of individuals with psychosis do not receive adequate mental health services (2). In the Hellenic context, despite decades of psychiatric modernization mandates aimed at deinstitutionalization and the establishment of community-based care, persistent challenges remain.

Specifically, the Greek mental health system suffers from chronic underfunding and a severe urban-rural workforce imbalance (3). While the nation boasts a high concentration of psychiatrists, their metropolitan concentration in major urban hubs creates significant service vacuums in remote provinces. This disparity is compounded by entrenched structural stigma, which drives a prolonged help-seeking lag: rural patients often present for their initial clinical encounter up to 15 years after symptom onset (4, 5).

This study focuses on the community of Kato Nevrokopi, a geographically isolated border region known as the “Siberia of Greece” due to its extreme winters and geospatial reclusiveness. Therefore, its population faces a syndemic pressure: systemic socio-economic stressors (e.g., unemployment, disability) interact with environmental precarity to create a high-vulnerability microcosm of psychological distress, framing Kato Nevrokopi as a purposive, high-stress “natural laboratory” (6, 7).

Community centers function as essential access points for these populations, providing critical care coordination and support aimed at combating systemic marginalization (8). Research indicates that these service users exhibit significantly higher rates of mental illnesses than the general population; previous studies have identified high symptomatic prevalence of anxiety (40%) and depression (17.1%) among rural women, highlighting the acute necessity for integrated behavioral health care models (9).

Mental resilience is defined as a dynamic process of positive adaptation following adversity, involving the fluid capacity to maintain mental health through cognitive flexibility (10). Similarly, meaning in life is a multidimensional construct comprising three pillars: coherence (making sense of life), purpose (goal-directedness), and mattering (the sense of existential significance) (11).

Existing literature confirms that mental resilience and meaning in life act as protective factors against affective distress (12). Conversely, loneliness and physical illness are recognized as global vulnerability markers (13). However, few studies have explored how these variables interact within a single, high-stress rural context defined by severe geographic and economic constraints.

This study aims to address this knowledge gap by analyzing the complex relationships between loneliness, mental resilience, meaning in life, and psychological distress among a cohort of 120 Kato Nevrokopi community center recipients. Therefore, the Kato Nevrokopi Community Center is not merely used as a site of observation, but as a place-based experimental model to investigate the interplay between isolation and mental health. By identifying mutable factors within this distinct socio-economic microcosm, we aim to establish a framework for precision interventions that are generalizable to underserved rural and border European communities.

## 2 Materials and methods

### 2.1 Study design and site justification

This observational study utilized non-probability sampling to analyze the mental health of 120 Kato Nevrokopi Community Center recipients. Recruitment and data collection were carried out from February to May 2025. The research framework received ethical authorization in late 2024 from both the Kato Nevrokopi Municipal Council (registry identifier: 11691/29-11-2024) and the Human Subjects Protection Board of the Hellenic Open University. Eligibility parameters mandated active service use and formal participant authorization. As the study utilized a cross-sectional, single-session design, no minimum duration of service exposure was required for inclusion, while zero participant attrition was noticed, with exposures and outcomes captured in a point-in-time assessment.

The study utilizes a place-based experimental model centered in Kato Nevrokopi, a geographically isolated border community known as the “Siberia of Greece.” This site was purposively selected to serve as a natural laboratory for investigating psychosocial predictors of mental health in extreme environments. By focusing on this high-stress, homogenous microcosm, we were able to isolate the impact of specific variables -such as the link between solitary residency and loneliness-while minimizing the confounding infrastructure variables typically found in multi-center urban studies. This methodological choice prioritizes high-fidelity data and internal validity, offering a “thick description” that ensures transferability to similar underserved European border regions.

### 2.2 Bias mitigation and internal validity

Several measures were implemented to minimize systematic bias: (i) Selection bias: Enrollment via a staggered protocol over a two-month period to mitigate volunteer bias; (ii) Interviewer bias: an unmediated self-reporting protocol was utilized, eliminating potential interviewer influence or interpretation bias; (iii) Measurement bias: Psychometric assessment relied exclusively on Hellenic-validated instruments (DASS-21, CD-RISC-25) demonstrating high internal consistency (*α* > 0.80); (iv) Confounding control: multivariable linear regression was employed, rigorously controlling for independent variables including age, gender, and physical illness history; (v) Reporting standards: This study followed STROBE guidelines for design and reporting (14).

### 2.3 Measures

#### 2.3.1 Socio-demographic information questionnaire

Participants were invited to provide anonymous demographic information on their gender, age, family status, history of chronic mental and/or physical illness.

#### 2.3.2 Connor-Davidson Resilience Scale (CD-RISC-25)

Participants completed the Connor-Davidson Resilience Scale (CD-RISC-25), a 25-item psychometric instrument designed to quantify psychological fortitude (15). Utilizing a five-point ordinal scale, aggregated scores are calculated up to 100, where elevated values reflect superior resilience levels. The Greek adaptation utilized in this study underwent methodological verification by Tsigkaropoulou et al. (16).

#### 2.3.3 Depression Anxiety Stress Scale-21 (DASS-21)

Participants completed the Hellenic validation of the “Depression Anxiety Stress Scale-21” (DASS-21) (17, 18), a 21-item affective distress inventory. Responses were captured on a four-point frequency scale, yielding subscale aggregate scores up to 42. In this tripartite assessment, results are positively correlated with the severity of psychological symptoms.

#### 2.3.4 Meaning in Life Questionnaire (MLQ)

Participants completed the Hellenic psychometric adaptation of the “Meaning in Life Questionnaire” (MLQ) (19), a 10-item bifactorial self-report inventory. Utilizing a five-point agreement scale, the instrument quantifies both the Presence of Meaning and the Search for Meaning. This tool was empirically validated for the local population by Pezirkianidis et al. (20).

#### 2.3.5 UCLA Loneliness Scale (UCLA-LS-20)

Loneliness was evaluated via the UCLA-LS-20, a self-report inventory pioneered by Russell et al. (21). Responses were recorded on a 1–4 Likert scale, with elevated aggregate scores signifying deeper levels of isolation. This study utilized the Greek version of the tool, which received methodological validation in 1992 (22).

### 2.4 Statistical analysis

#### 2.4.1 General approach and software

Data analysis was performed using SPSS Statistics 26.0, with the alpha level for statistical significance set at *p* < 0.05. Internal consistency reliability for all instruments was evaluated using Cronbach’s alpha coefficients (*α* > 0.80). The adherence of all continuous data to a normal distribution was evaluated via the Kolmogorov–Smirnov (K–S) test (Table 1). Most of study variables did not significantly deviate from normality (K–S *p* > 0.05), supporting the use of parametric statistics. Variables violating normality assumptions were analyzed using appropriate non-parametric tests. Detailed K–S statistics are presented in Table 2. The K–S statistic was applied uniformly across all DASS-21 measures, the psychosocial predictor scores (loneliness, resilience, and meaning in life), and age. Accordingly, parametric data are presented as Mean ± Standard Deviation (SD) and analyzed via Pearson’s correlation and independent *t*-tests. Non-parametric data are expressed as Median ± Interquartile Range (IQR) and were analyzed using Spearman’s *ρ* and Mann–Whitney *U* tests.

**Table 1:** Normality assessment using Kolmogorov–Smirnov test.

Variable	K–S statistic	*p*-value
Depression	0.081	0.200
Anxiety	0.094	0.087
Stress	0.076	0.200
Loneliness	0.063	0.200
Resilience	0.071	0.200
Meaning in life	0.069	0.200
Age	0.058	0.200

**Table 2:** Internal consistency reliability of study instruments.

Scale	Cronbach’s *α*
DASS-21 total	0.91
Depression	0.87
Anxiety	0.85
Stress	0.88
CD-RISC-25	0.93
MLQ – presence	0.86
MLQ – search	0.82
UCLA Loneliness Scale	0.89

#### 2.4.2 Data management and modeling

Complete Case Analysis was applied for variables missing completely at random (MCAR < 5%), while Multiple Imputation was utilized for more extensive missingness. Multivariable linear regression models were constructed to identify independent predictors of psychological distress, controlling for potential confounders including age, gender, and physical morbidity. To investigate whether physical health status moderates the relationship between loneliness and distress, an interaction term (Physical Illness × Loneliness) was included in a moderated regression framework, followed by simple effects testing.

#### 2.4.3 Variable transformation and grouping

To maximize statistical resolution, variables were analyzed in their continuous form within primary regression models. For descriptive and comparative purposes, data were subsequently categorized based on *a priori* clinical benchmarks: age was dichotomized at a 65 year threshold, and DASS-21 scores were grouped according to validated Hellenic severity categories. Living status was simplified into a binary variable to facilitate subgroup comparisons.

#### 2.4.4 Sensitivity analysis and validity

Several sensitivity checks were conducted: (i) Comparison of results between Multiple Imputation and Complete Case Analysis to ensure stability; (ii) Evaluation of influential outliers using Cook’s Distance; (iii) Verification of result invariance across different variable grouping strategies.

## 3 Results

### 3.1 Data quality and participant recruitment

The *N* = 120 dataset demonstrated exceptional completeness, with 0% missingness for demographic and health variables. Minor missingness was observed in the psychometric scales (0.8–2.5%). Recruitment at the Kato Nevrokopi Community Center involved screening 150 individuals. After excluding 15 for cognitive or eligibility reasons, 135 were invited to participate. One hundred twenty provided consent (92.31% participation rate), with no attrition during the assessment. Reasons for the 15 eligible individuals declining included time commitment (*n* = 10), privacy concerns (*n* = 3), and lack of interest (*n* = 2).

### 3.2 Sample characteristics

A sample of 120 participants (mean age 42.9 ± 13.1) was analyzed, with 69.2% being women. Most subjects were married with children, living in family units. Pre-existing physical conditions affected 15.8% of the group, whereas diagnosed mental illness was rare (1.7%). See Table 3 for descriptive characteristics.

**Table 3:** Participants’ socio-demographic characteristics.

Demographic variables	Frequency (*n*)	Percentage (%)
Gender		
Male	37	30.8
Female	83	69.2
Mean age (SD)	42.9	(13.1)
Marital status		
Single	31	25.8
Married	70	58.3
Divorced	14	11.7
Widowed	5	4.2
Having children		
No	37	30.8
Yes	83	69.2
Living arrangements		
Living alone	28	23.3
Living with family	92	76.7
History of mental illness		
No	118	98.3
Yes	2	1.7
History of chronic physical illness		
No	101	84.2
Yes	19	15.8

### 3.3 Levels of mental resilience

Resilience was measured on a 0–100 point system. Within this cohort, scores fluctuated between lower and upper bounds of 21 and 98. The central tendency for the sample was 64.6, indicating moderate to high levels of adaptive capacity.

### 3.4 Levels of depression, anxiety, and stress

The cohort’s affective distress markers showed low to moderate mean values across all domains. Depression, anxiety, and stress were within asymptomatic thresholds for most participants (45.1–77.1%). Moderate symptoms represented the secondary categorical distribution, whereas clinically acute (severe/very severe) presentations remained rare within this rural sample.

### 3.5 Levels of meaning in life

Participants’ meaning in life was measured on a 10–50 point scale continuum. The cohort exhibited a mean aggregate meaning score of 25.2 (SD = 6.3), with individual responses spanning from a minimum of 13.0 to a maximum of 40 points.

### 3.6 Levels of loneliness

Loneliness was measured on a 20–80 point scale continuum. The cohort exhibited a mean intensity of isolation of 41 (SD = 8.9), with individual responses fluctuating between a minimum of 24 and an observed maximum of 60 points.

### 3.7 The interplay of resilience, meaning in life, and loneliness

Meaning in life and mental resilience exhibited significant inverse associations with all dimensions of depression, anxiety and stress. Specifically, a robust meaning in life and mental resilience were protective factors against anxiety and depression. Conversely, perceived loneliness was directly associated with depression, anxiety and stress; augmented levels of loneliness consistently mirrored increased stress and anxiety. Bivariate correlation analysis (Table 4) confirmed these associations: loneliness was positively correlated with all dimensions of psychological distress, whereas resilience and meaning in life showed significant inverse correlations with depression, anxiety, stress, and overall distress. Furthermore, both meaning in life and mental resilience served to attenuate feelings of loneliness.

**Table 4:** Correlations between psychosocial variables and psychological distress.

Variable	Depression	Anxiety	Stress	DASS total
Loneliness	0.42***	0.39***	0.31**	0.45***
Resilience	−0.36***	−0.33***	−0.29**	−0.38***
Meaning in life	−0.41***	−0.35***	−0.28**	−0.40***

**p < 0.01, ***p < 0.001.

### 3.8 The impact of age, chronic illness, and living arrangements on affective distress

Older age and physical illness were key determinants of depressive symptoms. Advanced age was associated with reduced depression and anxiety, whereas individuals with long-term physical conditions exhibited an augmented affective burden compared to healthy counterparts. Furthermore, spousal partnership and physical illness were linked to heightened anxiety markers. Regarding social isolation, family-based domestic arrangements served as a protective factor, with residents in shared households reporting lower levels of loneliness than those in solitary residency.

## 4 Discussion

The purpose of the present study was to examine the links between loneliness, resilience, meaning in life, and psychological distress among community center recipients in a remote Greek region.

Consistent with existing literature, our results confirm that mental resilience and meaning in life serve as buffers against depression, anxiety, and stress (23, 24). These traits appear to provide individuals with the psychological resources necessary to navigate adversity without significant emotional decline. Conversely, loneliness emerged as the most significant risk factor for distress, showing a strong positive correlation across all measured dimensions. This underscores the critical need for interventions targeting social isolation, particularly for vulnerable groups like chronic patients or those living alone, who showed heightened risks for depression and loneliness, respectively (25, 26).

A central finding of this study is that older age acts as a protective factor against psychological distress. Older adults in our sample exhibited lower levels of depression, anxiety, and stress compared to younger participants. This aligns with the “aging paradox,” where emotional well-being often improves in later life despite age-related physical declines (27). This trend is often attributed to superior emotion-regulation skills and a lifetime of accumulated coping mechanisms (28).

However, our findings diverge from previous research in significant ways. While many studies suggest that older adults report higher mental resilience and a greater meaning in life (29, 30), our study did not find a significant association between age and these two factors. This discrepancy suggests that while older adults may experience less distress, this outcome may not always be driven by inherently higher resilience or meaning in life, but perhaps by other factors like reduced future-oriented worry or more stable social environments (31).

Therefore, our study revealed a complex relationship between marital status and mental health. While being married was associated with higher mental resilience-supporting many studies viewing marriage as a protective structure for well-being (32)—it was simultaneously associated with higher levels of anxiety. This unexpected finding challenges the conventional “marriage benefit” and highlights the unique stressors inherent in marital roles. Previous research suggests that such anxiety is often tied to relationship quality; marital discord, low spousal support, and traditional gender-specific burdens can transform a protective union into a source of chronic stress (33). Furthermore, our findings regarding marital anxiety may reflect a “selection effect,” where individuals with anxiety disorders statistically tend entering marriages, or an “emotional contagion” effect, where distress is shared between partners (34, 35).

Practical interventions include: (i) the implement of “value-centered” cognitive therapies to help individuals develop a “meaningful vital plan,” (ii) structured group activities that combat loneliness, (iii) incorporated psychosocial screening into primary care for patients with chronic physical illnesses, and (iv) local authorities and community managers engagement to create “safe spaces” that reduce mental health stigma. By integrating the proactive oversight of managers, the holistic support of community services, and the structural mandates of public policy, we can move from a reactive “crisis” model to a proactive “resilience” model. This study proves that even in the most isolated border regions, precision behavioral interventions can mitigate the impact of environmental and socio-economic stressors, provided they are supported by an integrated and well-funded mental health ecosystem. Accordingly, future research directions should be oriented toward: (i) “community resilience,” (ii) the long-term impact of “social prescribing” on meaning-in-life, and (iii) why specific rural populations engage with or avoid mobile mental health services.

## 5 Strength and limitations of our study

As an exploratory study in “Siberia of Greece,” this research provides crucial baseline data where none previously existed. It serves as a vital pilot that identifies specific local psychological needs, which can inform the design of future, more resource-intensive probability-based studies in remote Greek border regions. While these results offer valuable insights, they must be interpreted within certain limitations: (i) convenience sampling method and the relatively small sample size limit the generalizability of the findings. However, given the extreme geographic isolation of Kato Nevrokopi and the lack of a formal sampling frame for its most vulnerable residents, this method was the only feasible approach to reach this “hard-to-survey” group. Future research with probability-based sampling or multi-center designs should be utilized to validate these findings across diverse geographic and socio-economic settings; (ii) data collection was conducted through self-report tools, which may affect the accuracy and objectivity of the responses, as it is based on the participants’ assessment of their mental state; (iii) the cross-sectional design does not allow for causal inferences; (iv) the single-center setting may limit the generalizability of findings to other community-based environments; (v) due to the cross-sectional nature of the study, the identified predictors should be interpreted as associations rather than indicators of absolute risk or causality.

## Data Availability

The raw data supporting the conclusions of this article will be made available by the authors, without undue reservation.
